# Supplementary material for: Attention based automated radiology report generation using CNN and LSTM
Source: PLoS One. 2022 Jan 6;17(1):e0262209. doi: 10.1371/journal.pone.0262209 (PMC8736265; doi:10.1371/journal.pone.0262209)
Supplement: Attachment — Submitted filename: Response to Reviewers.docx [file pone.0262209.s001.docx]

**Editor’s Comments & Responses**

1. The proposed method needs to compare with other state-of-the-art methods, such as
2. <https://arxiv.org/abs/1912.08226>
3. <https://arxiv.org/abs/2010.10042>

**Response:**

**We have considered both articles and added some more relevant ones in revised version. We have also compared the results of proposed technique with these articles. Following recent articles have been added in revised version**

- **Alfarghaly, O., Khaled, R., Elkorany, A., Helal, M., & Fahmy, A. (2021). Automated radiology report generation using conditioned transformers. Informatics in Medicine Unlocked, 24, 100557.**
- **Liu, G., Hsu, T. M. H., McDermott, M., Boag, W., Weng, W. H., Szolovits, P., & Ghassemi, M. (2019, October). Clinically accurate chest x-ray report generation. In Machine Learning for Healthcare Conference (pp. 249-269). PMLR.**
- **Chen, Z., Song, Y., Chang, T. H., & Wan, X. (2020). Generating radiology reports via memory-driven transformer. arXiv preprint arXiv:2010.16056.**
- **Miura, Y., Zhang, Y., Tsai, E. B., Langlotz, C. P., & Jurafsky, D. (2020). Improving factual completeness and consistency of image-to-text radiology report generation. arXiv preprint arXiv:2010.10042.**

2. The experiments need to conduct on several datasets to demonstrate the model's generalizability.

**Response:**

**We have added one more publically available dataset for experimentations and results are added in revised article. We have added more results and also have highlighted failure cases to show model’s generalizability. Please refer to table-3, table-5 and figure 9.**

3. The paper needs to be proofread. There are many typos and errors.

**Response:**

**The article was initially proof read by a third party EdiTag. We have again proofread it for possible errors.**

**Reviewer's Comments & Responses**

1. **Is the manuscript technically sound, and do the data support the conclusions?**

Reviewer #1: Yes

Reviewer #2: Yes

2. **Has the statistical analysis been performed appropriately and rigorously?**

Reviewer #1: Yes

Reviewer #2: N/A

3. **Have the authors made all data underlying the findings in their manuscript fully available?**

Reviewer #1: Yes

Reviewer #2: No

**Response:**

**The datasets used in this article are publically available**

4. **Is the manuscript presented in an intelligible fashion and written in standard English?**

Reviewer #1: Yes

Reviewer #2: No

**Response:**

**Manuscript is updated according to the comments of the reviewers and academic Editor and all the grammatical and typographical errors are catered. It has been proof read by third party.**

5. **Review Comments to the Author**

**Reviewer #1**

The author proposed an attention based Automated Radiology Report Generation using CNN and RNN. The paper is easy to read and the results can confirm their idea. But there are still several issues that need to be addressed.

**Response: We are thankful to the reviewer for encouragement and also sharing his comments to improve the quality of our article**

(1) A line is missing at the bottom of the table.

**Response: We have updated the formatting of all tables and bottom lines are added at the end of all tables. We have also updated captions of all tables for better understanding.**

(2) The equation 2 is missing a ).

**Response: The equation is updated and ‘)’ is added at the end of equation 2.**

(3) The second Table 1 should be Table 2 and the Table 2 should be Table 3.

**Response: We have corrected table numbering.**

(4) The resolution of Figure 3 is low.

**Response: We have updated Figure 3 to improve its resolution. We have also improved the resolution of other figures.**

(5) The reference seems wrong. For example, in the section 4.3 Baselines:
    The proposed model is compared with many current best performing architectures, for example, LRCN [18] by Donahue et al., Soft ATT [19] by Xu et al., ATT-RK [20] by You et al., and Hieratical Generation [21] by Krause, et al. But in the Table 2 the references are 19-22. You have to check your references carefully.

**Response: References are updated and now all the references are properly aligned.**

(6) You need to include the latest method to compare with your proposed method, such as Meshed-Memory Transformer for image captioning, Linguistics Improving Factual Completeness and Consistency of Image-to-Text Radiology Report Generation. You can also compare with other methods, but at least those proposed in 2020.

**Response: As per reviewer suggestion, we have conducted more experiments and compared our results with more recent articles. We have also considered more public datasets like MIMIC for experiments and comparisons.**

**Reviewer #2:**

This manuscript describes an attention-based report generation from X-ray using CNN and RNN. Descriptions of x-ray images were missing from both data and experiment sections. More details on the images will be helpful (size of x-ray, divided image into n parts? what is n in the experiment?)

**Response: We have added these details with respect to both dataset and experimental setup in section 4.2 of revised article.**

VGG was not elaborated on Page 12.

**Response: Section 3.1 of revised article contains details related to VGG and we have also shown all layers of VGG in figures 2 and 3. The Softmax layer of VGG16 was replaced with the final 1 × 4096 FC layer. This layer now acts as an input to the decoder as well as the generation of medical reports. The output of the VGG16 network is a vector of size 1 × 4096, which will later be converted into a fixed vector length of 1 × 256 that is used to represent the features of the images.**

The experiments seem a bit confusing. For example, when LSTM and RNN were used in the experiment? Is it CNN-RNN or CNN-LSTM? In text, it is mentioned CNN-RNN, but in figure, it is CNN-LSTM.

**Response: Long Short-Term Memory (LSTM) networks are a modified version of recurrent neural networks (RNN) which makes it easier to remember past data in memory and considered best of the text generation tasks. The vanishing gradient problem of RNN is resolved here. So as a big picture we write RNN every where even in the title of the paper. But actually, we are using the best model of RNN which is LSTM. But as highlighted by the reviewer, we have replaced RNN with LSTM wherever we are mentioning proposed technique. We have also updated the title accordingly.**

The text of the manuscript can be reduced significantly.

**Response: As advised by the reviewer, we have reduced the text in introduction and literature review sections. However, more results and discussions are added to emphasize more on proposed system robustness. So in general the length of article is almost same.**

Please provide all the parameters for all the algorithms.

**Response: We have added parameters in figure 2,3 and 4 of revised article. We have also added table 2 to show hyper parameters.**

Captions for images are not available.

**Report: As per journal formatting, we didn’t add figures in the text. However the image captions are added at relevant places for better understanding.**

Problems in table caption. The numbering of tables is wrong.

**Response: We have corrected table numbering and have also improved the captions for better understanding.**
