# Supplementary material for: Attention based automated radiology report generation using CNN and LSTM
Source: PLoS One. 2022 Jan 6;17(1):e0262209. doi: 10.1371/journal.pone.0262209 (PMC8736265; doi:10.1371/journal.pone.0262209)
Supplement: Attachment — Submitted filename: Response File.docx [file pone.0262209.s002.docx]

**Comments & Responses**

**Comment:**

Please briefly describe the baseline methods

**Response:**

**We have added brief description of baseline methods in section “4.3 Baseline” of revised article.**

**Comment:**

Please check figure 4
**Response:**

**Figure 4 is updated. Now it is properly visible.**

**Comment:**

Please make sure the codes are publicly available.

**Response:**

**We have made our complete code publically available at our research group website. It can be accessed at** [**http://biomisa.org/index.php/downloads/**](http://biomisa.org/index.php/downloads/)
