# Supplementary material for: Attention based automated radiology report generation using CNN and LSTM
Source: PLoS One. 2022 Jan 6;17(1):e0262209. doi: 10.1371/journal.pone.0262209 (PMC8736265; doi:10.1371/journal.pone.0262209)
Supplement: Attachment — Submitted filename: Response File-II.docx [file pone.0262209.s003.docx]

**Comments & Responses**

**Comment:**

Figure 9 on page 39 is black

**Response:**

**We have updated the figure.**

**Comment:**

Figure 4 on page 40 is black
**Response:**

**Figure 4 is updated.**

**Comment:**

I feel the table on page 12 is not Fig 4.

**Response:**

**We have updated this**
